# Radon Gas in the City of Alicante. High Risk of Low Indoor Air Quality in Poorly Ventilated Buildings

**DOI:** 10.3390/ijerph17238762

**Published:** 2020-11-25

**Authors:** Carlos Rizo-Maestre, Víctor Echarri-Iribarren

**Affiliations:** Department of Building Construction, University of Alicante, Carretera San Vicente del Raspeig, s/n, 03690 San Vicente del Raspeig, Spain; Victor.Echarri@ua.es

**Keywords:** underground building, heritage building, radon gas, quality air, ventilation, healthy architecture

## Abstract

In December 2019, Spain considered for the first time the presence of radon to the Technical Building Code (Basic Document HS 6: Radon Exposure Protection), although it only mentions minimum presences and the need for ventilation. This research shows that in buried structures or in places with little ventilation, even in soils with a low probability of granite, a high content of radon gas can be found. The city of Alicante has been used as a measurement location for different architectural sites; here, the level of 100 Bq/m^3^ is the first threshold where the gas must be monitored, and the level of 300 Bq/m^3^ is the maximum threshold above which corrective ventilation measures must be taken. The research conducted during the years 2015 and 2016 shows that it is necessary to account for also the areas considered to be “low presence of radon gas” to achieve healthy constructions. The renewal of air in the different places will be tested for the presence of radon, i.e., the greater the accumulation is, the less ventilation and the greater the risk of accumulation of radon gas. This study is located in the city of Alicante, where the seven civil constructions are located: two Civil War shelters, the Santa Barbara Castle, the Ereta Powder Keg, the Luceros-Marq and Serra Grossa railway tunnels and the Británica underground deposits. Radon gas is currently a concern for major health and medical agencies because it is considered to be a chemical element that is very harmful to people. The World Health Organization is one of the organisations that has the objective of studying and researching this element, to develop solutions. Radon gas is normally found in a gaseous state and is highly radioactive. It is present in many terrains and it is mostly found in those with granite; although the presence of this element is very low, there is always a minimum presence. In the past, in nongranite soils, the dose of radon was considered to be so low that it was insignificant. Therefore, in this research, the aim is to consider the high presence of radon gas in nongranite soils as long as the conditions for its accumulation are present.

0pt plus 1fil0pt plus 1fil

## 1. Introduction

This research is based on the premise that we must attempt to demonstrate the need for ventilation in buried structures, even in places that have little presence of radon gas. To accomplish this goal, the city of Alicante is used as a measurement site for buried buildings (which have a higher risk of radon due to contact with the ground). The city of Alicante is located in a radon exposure category of 0, which according to information provided by Marna, means low exposure [1]. This category is most likely the reason why there is hardly any data on measuring the radon content in buildings in Alicante.

This work studies the air quality in certain places within the historical and civil architecture of the city of Alicante. Specifically, radon measurements have been made in the historic quarter of the city (Civil War shelters), Mount Benacantil (Santa Barbara Castle with several rooms dug out of the rock itself, La Ereta Powder Magazine and the Luceros-Marq railway tunnel) and the Serra Grossa (La Británica underground deposits and the Serra Grossa railway tunnel).

The measurements assume the possible need for improved ventilation to achieve better indoor air quality in buildings that are underground or in close contact with the ground, using radon gas as a quantitative indicator.

Radon gas can be measured actively or passively. The active measurements require electricity and can continuously collect data on the radon gas concentration and its variations over time during the measurements [2]. The passive measurements do not require electricity for the data collection [3].

In this investigation, ionic electrode chambers, or ICDs, were used. These are passive systems that detect radon with a series of detectors that are integrated into an internal element called an electret. The function of the electret is double: it can act as an electric field generator and a sensor in an ion chamber that is a vessel of known volume. The chamber has a filter that allows the gas to enter by diffusion and prevents the entry of the other elements produced in the disintegration process [4]. Inside the chamber, therefore, the radon disintegration takes place, and the emitted radiation makes the detector surface voltage decrease [5]. The analysis ends by relating this voltage drop to the concentration of radon in the studied time and space by means of a calibration factor.

In underground constructions or ground floor buildings with a large percentage contact with the ground, the most important source of radon gas is the ground, due to the radium that is present in it. The radium is normally found in the soil in concentrations between 10 and 50 Bq/kg, although it is possible to reach much higher values. Normally, the average value is considered to be 40 Bq/kg, and high or low concentrations as limits are established from this average value. Therefore, the amount of radon gas inside a building in contact with the ground depends on two variables that concern the radium-226 that is present: its concentration and the permeability of the ground itself for the passage of the gas.

## 2. Methodology

### 2.1. Radon Gas as a Ventilation Indicator

Radon gas, symbol Rn (Rn222 isotope), is element number 86 on the periodic table and falls within the group of “noble gases”. In different soils, the presence of uranium and its decomposition disintegrates and produces radium, which in the next process is released as gas [6]. Radon gas cannot be detected by humans due to its gaseous form, which makes it colourless, odourless and tasteless [7].

Because many rocks contain uranium, they produce radon gas at the end of their chain in the process of disintegration [8]. The gas released by the ground and by building materials accumulates in enclosed places, such as underground buildings and natural caves [9]. Therefore, it is considered to be very important that buildings are properly ventilated regardless of their use [10].

Radioactivity is produced in the environment and can be caused by different factors, although 75% of it corresponds to natural elements [11]. One of the largest sources of radioactivity in the natural environment is radon gas, which generates a major public health problem because it is present in many places, mainly in buildings and drinking water, and therefore, it must be accounted for and studied [12].

Ionizing radiation is the process by which radon is disintegrated, and when it enters matter, it removes the electrons from the surrounding atoms through the process of ionization [13]. When the matter is biological tissue, which has a high water content, ionization produces free radicals that have high chemical activity [14], and they produce molecular alterations in the tissues of living beings [15]. One of the most important alterations is chemical changes in DNA, which is the basic organic molecule that makes up the human body [16]. These changes can produce abnormal cell development and other biological effects. Depending on the dose of radiation received, these changes can be more or less severe, i.e., the more radiation there is, the greater the risk of serious changes. Lung cancer is the main effect produced in humans by the presence of radon and contact with it [17]. Radon, a gaseous radioactive element, is present in the various construction materials used in buildings and in the soil where they are installed [18].

The Bequerelium cubic meter (Bq/m^3^) is the unit of measurement used to measure the presence of radon gas in closed places, i.e., the concentration per unit volume. The conclusions of this work have been developed on the basis of the recommendations of different national and international organisations, among them the European Union, the WHO and the Nuclear Safety Council (the only competent body in Spain for nuclear safety and radiation protection), which establish the thresholds for the presence of radon and the limits above which corrective measures should be taken. In this investigation, 100 Bq/m^3^ is considered to be the first threshold above which the presence of the gas should be monitored, and corrective ventilation measures should be taken above the threshold of 300 Bq/m^3^.

Radon gas is harmful to human health and is a highly carcinogenic element. Therefore, new building regulations are working to incorporate this point as a control element. The main source of this gas is the land, more specifically, the constructions that have low measures of protection or underground areas that are susceptible to accumulating greater amounts of this gas. New constructive forms make the buildings more and more hermetic and prevent the renovation of the inside air. Other factors to account for are the temperatures when they are extreme, since they facilitate the accumulation of the gas by differences in the pressure, and the precipitations that will favour ionisation in the places of concern.

### 2.2. Prediction of Radon in the Ground

The most direct and reliable method to identify the areas likely to have high concentrations of radon is the production of maps from measurements of its concentration in the indoor air of homes. This method requires a very high number of measurements throughout the territory, so alternative indirect methods have been developed that use other magnitudes correlated with the concentration of radon in homes. Since the main source of radon in a building depends on the terrain on which it stands, the concentration of radon in the gas phase of the soil is a good indicator. This variable in turn depends on the radium-226 content of the soil and the underlying rock, as well as the degree of fracturing or weathering of the rock formation and the permeability of the soil.

The radon gas content in different countries has been studied according to previously established parameters, which are usually a function of the igneous concentrations [19]. Sweden has mapped radon gas concentrations in the ground at a depth of one meter [20,21]. Other countries have tested these methods with other variables, such as the soil concentration of radium-226 or its equivalent in uranium. In France, there is a geological map of the average uranium content per geological unit [22]. Germany [23] has also developed a map of geogenic radon gas potential, as has the Czech Republic [24]. Predictive maps of radon gas are based on the premise that granitic soils are at the highest risk of radon content due to their concentrations [25]. Clay soils are considered to be low in radon gas. At the end of 2019, the Spanish Technical Building Code, or CTE, established the maximum recommended radon content and how to act on the different values.

In Spain, the most valuable source of environmental radiological information is the Map of Natural Gamma Radiation (Marna), which evaluates the rate of exposure to terrestrial gamma radiation of natural origin referred to 1 meter above ground level, which has an excellent correlation with the content of radium-226, which is the precursor isotope of radon. Therefore, Marna is a predictive map of the amount of radon in the ground [1].

The Spanish technical code establishes a reference level of 300 Bq/m^3^ for the average annual concentration of radon in the interior of habitable premises in order to limit the risk of exposure of users to inadequate concentrations of radon coming from the ground [26]. This value has been taken into account in the study as a barrier where urgent measures have to be taken. A second level of control has been established around 100 and 300 Bq/m^3^ to monitor the different sites.

### 2.3. Measuring Equipment: Ionic Electret Chambers

The equipment used to carry out the measurements of the study was the Ionic Electret Chambers (IEC), passive devices that work as integrating detectors to measure the average concentration of radon gas during the measurement period. The electret works both as a generator of an electric field and as a sensor in the ion chamber. The radon gas, but not the decay products, enters the chamber by diffusion through an inlet equipped with a filter. The radiation emitted by the radon and its disintegration products formed inside the chamber ionizes the air contained inside the chamber by reducing the voltage of the detector surface. Subsequently, a calibration factor relates this voltage drop to the radon concentration in the space under study.

The accuracy certified by the manufacturer is close to 10 per cent of variation in the different measurements, provided that it is used properly. The chambers function as a container of known volume that relates the amount of radon capable of modifying the state of the electret in a given time. Therefore, with this method we can relate the radon present inside the container to that present in the room where it is deposited. The types of cameras used for the study are short term, as are the electret ones. These cameras are used for measurements between 1 and 5 weeks. Cameras and electret are usually used for longer measurement times.

## 3. Analysed Buildings

**Figure 1:**
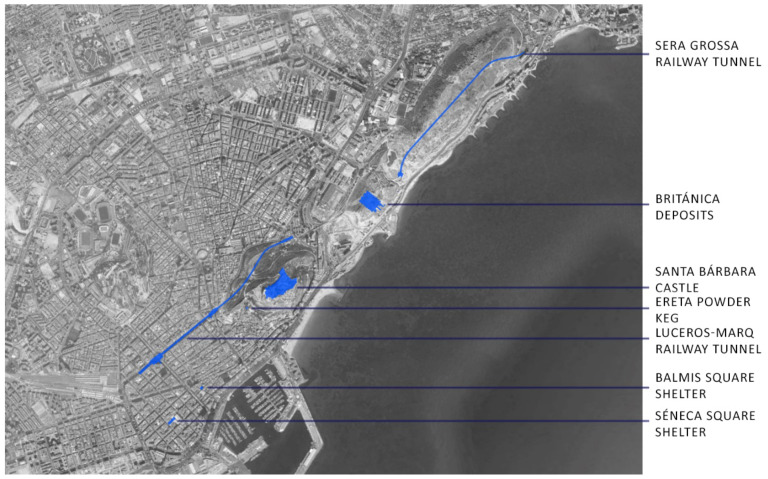
Image of the different enclaves chosen in the study of the city of Alicante: the shelters of Plaza Balmis and Plaza Seneca, the Castle of Santa Barbara, the Powder Magazine of the Ereta, the deposits of the British and the two tunnels of the TRAM of Alicante (Source *Google Maps*. Own elaboration).

This article aims to measure the amount of radon gas present in the Mediterranean coastal city of Alicante. To do so, civil buildings from different periods and typologies must be measured. The first buildings to be measured were the Civil War shelters in the city of Alicante, which were built for the passive defence of the city, and they have now been restored and can be visited; these are the shelter in Plaza Seneca and the shelter in Plaza del Dr. Balmis. Next, we describe three more buildings that were studied, which are the most important on Mount Benacantil. First is the Santa Barbara Castle, which was built on a rock at an altitude of 170 m, from which all of Alicante Bay can be seen; this castle is an icon of the city. Second is El Polvorín de la Ereta, which is located in the park of the same name and which contains important historical information about the enclave. Third is the Luceros-Marq railway tunnel, which is also linked to Mount Benacantil [27]. The final part of the study is dedicated to two civil structures that are related to the Sierra de San Julián: the railway tunnel, which was excavated in the rock itself, and the underground deposits of the Británica, which were used by the CAMPSA company to store and distribute oil derivatives until 1966 [28]. At the time that the study was conducted, the section of the Sierra de San Julián rail tunnel was not yet open for tramway use. The deposits of the Británica have an entire underground level dug into the rock. As a result, these are two good examples for checking the supposedly higher radon content due to the structures being the buried and unused type. Figure 1 shows the locations of these seven buildings on the map of the city of Alicante.

The city of Alicante, located on the southern Mediterranean coast on limestone rocks, is considered to be a low or very low risk area within the Marna map of exposure to radon gas, and therefore, the most important research teams have not traditionally focused their attention on it. In this research, the objective was to study the historical and civil architecture in the city of Alicante while considering radon to be an element that forms part of the constructions, to determine whether preventive measures should be taken due to the accumulation of this gas inside them.

In the choice of architectural structures for this study, both old (Spanish Civil War shelters, Santa Barbara Castle, the Ereta Powder Magazine and the British Embassy depots) and current constructions (Luceros-Marq tunnel) or those under construction (Serra Grossa tunnel) have been considered. From a geographical point of view, these actions are located at three strategic points of the city: the Urban Centre, Mount Benacantil and the Serrra Grossa. In all of the structures studied, a review of their historical and constructive context has been conducted prior to the detailed measurement of the air quality with radon gas as an indicator in each of them.

The two Spanish Civil War shelters that have been part of this study are considered to be historical constructions, and they are located under two urban squares in the city of Alicante, Seneca and Balmis.

The Castle of Santa Bárbara, with more than 1200 years of history, dominates the city of Alicante from the top of Mount Benacantil, with a height of 166 m, which has historically made it the most important strategic point on the Alicante coast. On the mountain, the Polvorín building can be found, located in the Parque de la Ereta, and it is a construction from more than 200 years ago that still retains almost all of its original design.

In the two most important mountains of the city of Alicante, Monte Benacantil and Serra Grossa, there are two tunnels of contemporary development that have been included in this study due to their integration in the terrain. These mountains are located in the northern part of the Betic Mountain Range and are characteristic for their rock, the San Julián Stone.

In the Serra Grossa, there is also a very important engineering structure from the 20th century that is unknown to the inhabitants of the city, namely, the deposits of the Británica, which has a footprint excavated in the mountain of 20,000 m^2^ and domes of approximately 20 m high. At present, it is in a state of abandonment, and there are incipient initiatives to convert it into a cultural enclosure.

Modern architecture increasingly incorporates more technological advances in both its designs and types of material. For this reason, the current objectives of architecture are not centred on only the distribution of space because other factors are considered, such as the thermal behaviour or the degree of insulation with external factors. Radon gas is presented as a radioactive gas that is present both in the construction materials and, above all, in the terrain. Therefore, it is necessary to provide buildings with crash barriers that prevent the entry of the gas produced by elements outside the building construction.

The evolution in architecture developed in recent years has caused new constructions to have better protection, smaller natural ventilation and greater energy savings, which makes the atmosphere inside the buildings more and more closed and hermetic, and therefore, the concentrations of radon gas are increased more and more when it cannot escape and must instead accumulate. In this way, basements, garages and other areas in direct contact with the outside are likely to reach higher values of concentrations of this gas because the main source of radon in nature is the ground [10].

### 3.1. The Plaza Seneca Shelter

This refuge from the Spanish Civil War is a civil building for defensive use with architectural characteristics of buried concrete slabs, as the municipal architect of the time left a reference in his archives of 9 March 1937 [29].

Francisco Lozano Olivares and Marcos Lumbreras Voigt conducted an investigation in 2015 on the refuge and its restoration, in which they studied the structure and its foundations, which were buried under the buildings that were demolished in the subsequent construction [30].

**Figure 2:**
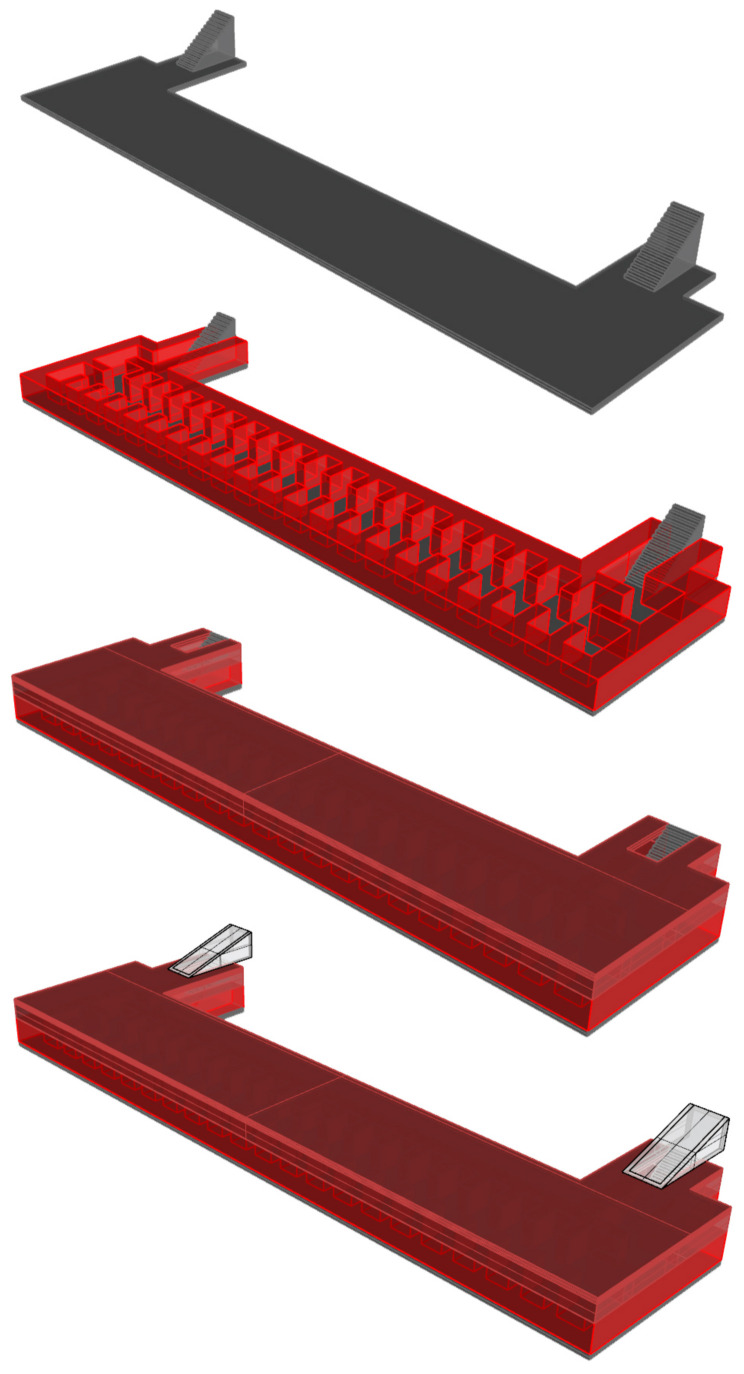
Image of the 3d reconstruction of the entire volume of the shelter.

The shelter is composed of a single rectangular body volume of 51 by 12 m, in addition to the two lateral and symmetrical entrances on the 9-m bottoms (Figure 2). The project was conducted while accounting for the large size of the piece, and therefore, the foundation slab is composed of a variable concrete frame of between 60 and 180 cm [31].

**Figure 3:**
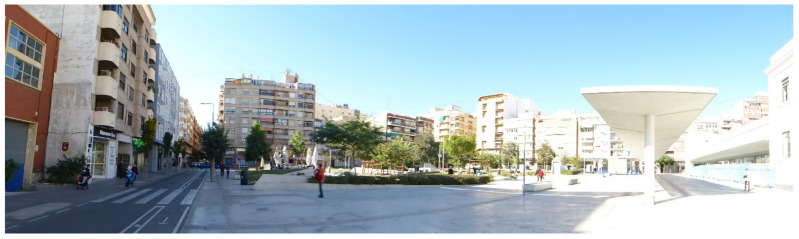
Current view of Seneca Square.

The shelter had 65 ceramic vents, which ranged from 7 to 140 centimetres; they pierced the concrete slab and ventilated the inside of the shelter along the central corridor that connected with the 38 cubicles. The layer following the concrete slab was of compacted earth to cushion the effect of the pumps. Therefore, the only natural ventilation entrances were the ventilators and the entrances to the shelter. Figure 3 shows the current appearance of Seneca Square where the shelter is buried.

### 3.2. The Plaza Balmis Shelter

**Figure 4:**
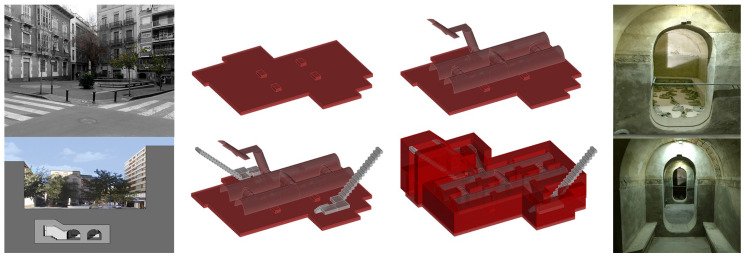
Plan of the Plaza Balmis shelter, with the six cells connected in both directions forming two large longitudinal naves. The lateral images show the access and section of the square (**left**) and the interior of the refuge (**right**). (Own fountain).

The refuge of Plaza Balmis is of the cellular type, according to the characteristics of the municipal architect of the time, and the body of the refuge is buried two meters above the level of the square. The volumetric layout of the refuge is composed of six rectangular cells with an approximate surface area of 7.5 m^2^, each one with a height of between 1.81 m and 2.33 m. The six rooms are connected by steps of approximately 1 m. The whole of the refuge has approximately 50 m^2^ without counting the accesses (Figure 4).

The construction has two zigzag entrances at the end of each staircase, to attenuate the effects of the shock waves caused by the bombs, and in case one of them was impossible to use, the other was the escape route for the occupants of the shelter. The accesses are built on a mass concrete base that serves as a support for the sections of stairs built with hollow brick. The interior access to the square, which corresponds to the intersection of Limones and Cid streets in the city of Alicante, is currently walled up due to the different actions that have been conducted in the square. The walled access was probably filled in with filler from the demolition of the roof, which was conducted in 1946 [32].

The upper enclosure is made of hollow brick, which was placed in the form of barrel vaults with lowered arches and, then, arranged with tambourines and plastered. The upper end of the vaults is made of reinforced concrete as in other similar fortifications, such as the Alfonso El Sabio or Plaza de Campoamor shelters, which were demolished in 2007 to build the Alicante Provincial Council Auditorium (ADDA). The pavement is made of mass concrete filled with compacted sand.

### 3.3. The Castle of Santa Barbara

The Castle of Santa Barbara is situated in the most exceptional area of the city of Alicante, where its height is 166 m above sea level and its distance from the coast is 200 m in a straight line, which make it the most important strategic point of the coast of Alicante. This situation allowed the occupants of the castle to glimpse the enemy’s approaches both by land and by sea.

**Figure 5:**
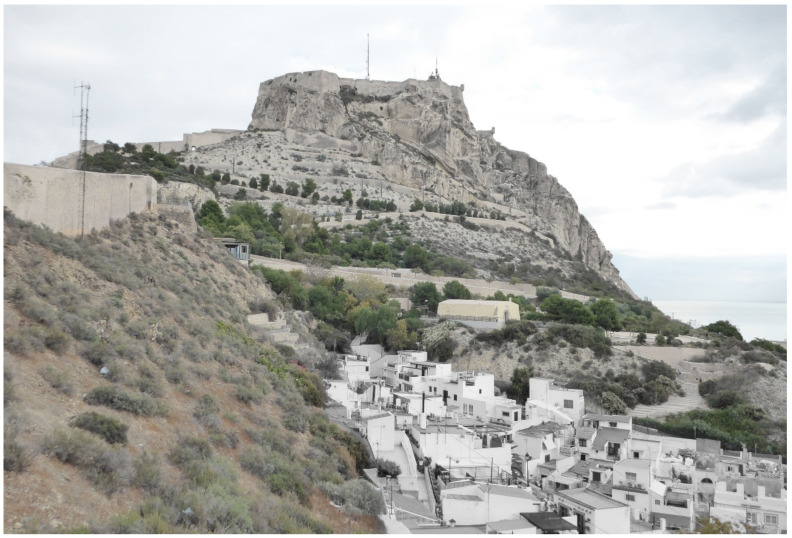
Image of the Castle of Santa Barbara from its southern side, recognised as the “face of the Moor”, on the same slope where the Ereta magazine is located.

The castle is located at the top of Benacantil Mount, a rocky mountain; from the viewpoint of the beach, it has an appearance that is called “the face of the Moor” because of its morphology (Figure 5). This mountain has been the object of archaeological work where remains have been found from the Bronze Age, the Iberians and the Romans, which shows the importance that it has always had for the city [26].

The name of Santa Bárbara Castle was obtained when it was conquered by the Infante Alfonso X El Sabio from the Arabs on 4 December 1248, the day of the feast of Santa Bárbara [33]. In 1296, after several disputes over its ownership, it was conquered by the Crown of Aragon, and its refurbishment was ordered. Since then, many kings have ordered remodelling work to modify the aesthetics of the castle, until Philip II conducted the last major remodelling work, which has lasted to the present day, since it has only been subject to conservation work. These structures lasted between 1562 and 1580, with projects by Juan Bautista Antonelli and Jorge Palearo “El Fratín”.

The city of Alicante has been attacked at different times, which has also affected the conservation of the castle. In 1691, it suffered some bombardments, caused by the French army, which affected the whole enclosure. In the War of Succession during the period 1706–1709, the castle was in the hands of the English and suffered great damage. The military action that caused most of the damage to the fortification was in 1873 when the armoured frigate “Numancia”, in the hands of cantonalist rebels from Cartagena, launched a bombardment on the city of Alicante.

In 1963, it was opened to the public after many years of neglect and the wear and tear of the Spanish Civil War. The castle was equipped with two lifts from the Avenida Jovellanos, parallel to the Paseo del Postiguet, to facilitate the climb to the mountain, with a height of 142 m.

### 3.4. The Ereta Powder Keg

**Figure 6:**
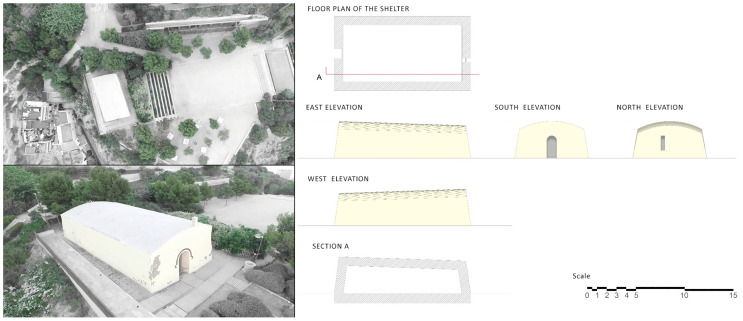
Image of the Ereta powder magazine made with a drone at a height of 20 m above ground level.

The powder keg de la Ereta is a construction that is located in the Parque de la Ereta, on the western slope of Mount Benacantil, and it served as a defensive fortress to store weapons for the protection of the city [34] (Figure 6).

This structure is a ship from century XIX that is very well-known by the alicantina population given its privileged situation, but there is little historical route documentation that shows the importance of this construction within the alicantina history. Information on this building is very scarce due to the passage of time and the atypical nature of the place [35].

The powder keg is a rectangular building with a single height, with the roof in the form of an arch, generating a slight slope. This construction is very thick, approximately one meter on all sides due to its security and defensive character. As a peculiarity of this type of place, its constructive typology made its walls structurally stronger than its roof to control the expansive wave in case of an explosion.

During the Islamic occupation of Alicante, between 711 and 740, the population grew, and it became necessary to protect it from possible attacks by invading armies. The city was protected by a wall that surrounded it completely and had the main access in the Puerta Ferrisa, which is located in what is now known as Calle Mayor and Calle Torrevieja, until it was demolished in 1860 [36,37].

### 3.5. The Luceros-Marq Rail Tunnel

**Figure 7:**
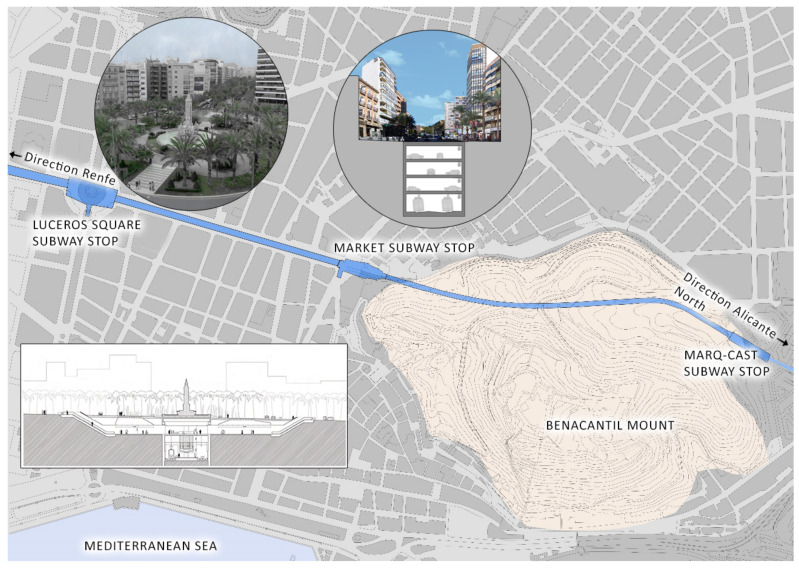
Location map of the Luceros-Marq rail tunnel.

The Luceros-Marq railway tunnel is, together with the Serra Grossa tunnel, one of the most important civil structures built in the 21st century in the city of Alicante. This infrastructure connects the Marq-Castillo stations, at the foot of Monte Benacantil, with the Luceros station, although in the future, its aim is to reach the Renfe railway station itself and serve as a connection to the city, with travellers from all over Spain. The route of the tunnel passes under the avenues of Alfonso X el Sabio, de la Estación and Jaime II until it enters the Monte Benacantil, reaching a height of fifteen metres below the zero level of the street (Figure 7).

The tunnel started as one of the initiatives of the Valencian Community in the field of transport on 10 November 1986, when the public law entity Ferrocarrils de la Generalitat Valenciana was constituted, which depended on the Department of Public Works, Town Planning and Transport, with the intention of developing suburban trains, trams and metropolitan railways. In 1988, the metro was inaugurated in Valencia, and four years later, the tram began its development in Alicante.

The planning of Alicante’s Metropolitan Transport (TRAM) is part of the *2004–2010 Strategic Infrastructure Plan*, which has the aim of improving the city’s mobility system. Alicante was a city with a history of trains; although the tram had disappeared thirty years earlier, it still had the “trenet”, the train that connected the entire northern coast of Alicante with the centre.

The railway infrastructure that in the future will connect the Renfe station with the northern area of Alicante had the problem of passing through one of the most important avenues (Alfonso X el Sabio), where the road traffic is very dense in the middle of the city centre. The project ends up raising the idea of burying the tunnel and providing the city with parking in these large avenues.

Based on the idea of burying the tunnel and marking the areas where the train would pass, in July 2005, the Regional Ministry of Infrastructure and Transport commissioned the Alicante architect Javier García Solera to design the new underground station for the TRAM at Luceros, which would serve to connect the route from the Santa Bárbara Castle stop with the city centre by means of a tunnel [27]. This station is located below the square from which it takes its name, between the market stations and the future Renfe station, which will be an intermodal station for the city, housing both the metropolitan train and the long-distance train.

The Luceros station was built in the centre of the square and was located 22 m below level 0 of the square. The entrance to the TRAM stop is on level −1 and can be reached by stairs or an elevator, which was the only element that modified the street level [27]. The train passes at level −4 and adapts to the slopes of the street until it enters the slopes of Mount Benacantil. Levels −2 and −3 correspond to a car park that serves the avenues Alfonso X el Sabio and Jaume II, which is only interrupted at the TRAM, Luceros and Mercado stops.

### 3.6. The Serra Grossa Railway Tunnel

**Figure 8:**
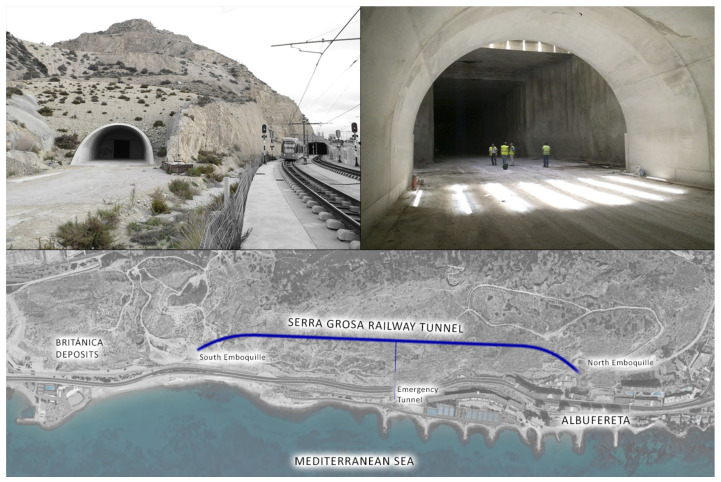
*Google Maps* image of the Serra Grossa with the location of the two infrastructures studied. (Own elaboration).

The Serra Grossa railway tunnel is a modern civil infrastructure that forms part of the project to renew the route of the Alicante train lines. This construction belongs to the railway variant of the L1 line in the section of the Finca Adoc, which connects the city centre to the northern part of the province of Alicante, passing through El Campello, Villajoyosa, Benidorm or Altea (Figure 8).

Work on this tunnel began in 2008 but was halted years later due to a lack of liquidity to address the end of the work until the present day. As a result, the excavation is almost complete in the absence of the entrance or north portal. Until the completion of the work, the line that runs northward from the centre of Alicante takes advantage of the corridor of an old FEVE line, which in the future is to be fitted out and equipped as a green area within the project for “Landscape and urban integration of the tramway as it passes through the Serra Grossa”.

The Serra Grossa tunnel has a total length of 1470 m, of which 30 m are a false tunnel at the south portal, 1315 metres are a tunnel in the mine, built with a T.B.M., and 125 m are a tunnel at the north portal between screens using the cut-and-cover system. This technique, also called false tunnel, is used when the surface is very close and the sides must be reinforced with piles before concreting [38].

The mine tunnel has a standard section with a 4.67 m inner radium and straight gables 2.39 m high. The free section inside the tunnel is 50.5 m^2^ and has two evacuation passages, with a useful width of 1.20 m.

There is an evacuation gallery or emergency tunnel, which is perpendicular to the main tunnel, and it is 144 m long and 12 m^2^ in the section, which runs from the interior of the tunnel to the current railway platform, with a future pedestrian walkway.

The terrain in which the tunnel is located corresponds to bioclastic calcarenites with variable foundations and tertiary marls that have undergone some tectonic processes. These tertiary loams correspond to materials with worse geotechnical characteristics, which are found at the end of the tunnel in the mine, along a small section that is 90 m long [39].

### 3.7. The Británica’s Deposits

**Figure 9:**
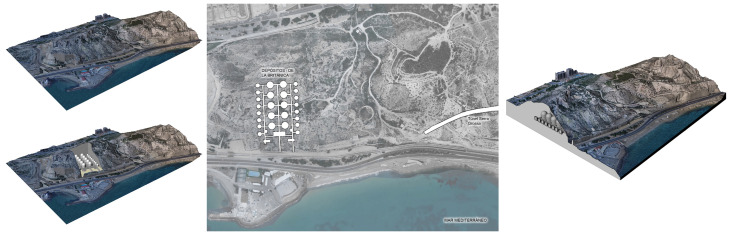
Image of the locations of the deposits on the Serra Grossa with a drone flight.

Inside the Serra Grossa is one of the most important engineering works of the 20th century, which is unknown to almost all of the inhabitants of the city: the storage domes of an old oil refinery that was initially owned by the state and later by the private company Campsa (Figure 9). This infrastructure served much of the country until 1966, when a more modern structure was inaugurated in the port of Alicante.

This industrial facility was used for the transformation process of oil and its derivatives, from storage to distribution. The facility had both surface and underground constructions dug out of the rock of the Serra Grossa. This complex was connected internally with galleries and tunnels at different heights that communicated the interior rooms with the exterior rooms.

Despite the deterioration of decades, it is still possible to see part of the complex, especially the part that remains buried in the mountain. Some of the elements of the old factory remain inside; wagons, rails and electric cables are found in the dirt, and there was complete abandonment.

The study of the indoor air quality using radon gas as an indicator inside this complex is presented not only because of its interesting architectural morphology, being built in a mountain, but also because of the importance it has in the history of the city and the little knowledge the population has of this characteristic place.

Apart from the underground volume that makes up the complex today, there are few elements that were once part of such an important industrial factory in the city. Today, it is abandoned and, due to its size and historical route, it can be considered to be part of industrial archaeology [40].

The enclosure was composed of three main galleries and seven secondary galleries, which were linked in a grid pattern, and they housed large concrete vaults on their sides in which metal tanks were installed to store the crude oil. The main vaults are approximately 20 m high and 18 m in diameter. The communication galleries have an average section of 3 meters by 2 m in height and a length of almost 160 m, with an arrangement of 10 main and 16 secondary vaults. The total footprint of the underground complex is located in a rectangle of 170 by 120 m.

The main entrance to the complex was the central high gallery where maintenance and repair work was conducted. The fuel tanks were filled by means of large pipes that came to the surface and connected with the external distillation and crude oil handling plants.

On the lower level, there were different rooms, such as warehouses, workshops, offices, garages, storage rooms and even workers’ houses. This whole area was demolished, and now, the Alicante Metropolitan Transport stop of La Sangueta is located there.

On the next level was the large exterior tank, which is still partially standing, and the chimney, which can be seen in the photos of the time. There were also old exterior gabled halls with masonry walls.

As described above, the complex has been in disuse since 1966, and no work has been conducted to reconvert or clean up the site. The refinery has been used for film making, but it has not been put to any further use due to the low level of conservation over the past 50 years. Various proposals have been made to rehabilitate the complex, including a proposal for a leisure and cultural park that would take advantage of the underground enclosures and its high domes as an auditorium area.

The Special Plan for the Archaeological Protection of the Municipality of Alicante includes the deposits, but it does not provide any type of protection. A report by the Directorate General of Heritage of the Regional Ministry of Culture of the Community of Valencia recommended that the facilities be declared a “Site of Local Importance”.

On the occasion of the study of the route for the new metro line that crossed the Serra Grossa to the north, the possibility was considered of crossing the factory as part of the excavation of the tunnel, which would have meant the total loss of the site. This idea was ruled out by the Regional Ministry of Infrastructure and Transport, which made its subsequent rehabilitation possible, making recovery plans similar to those mentioned above viable.

## 4. Results

### 4.1. Plaza Seneca Shelter

**Figure 10:**
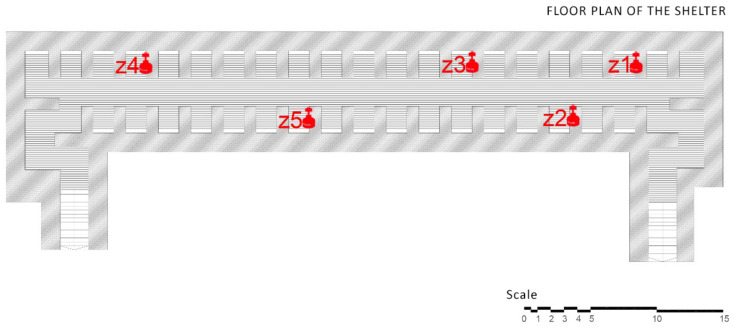
Measurement areas made inside the shelter in Seneca Square.

The data collection phase in the study was conducted at the Plaza Seneca shelter, and measurements were taken between 18 and 28 January 2016. This process covered the entire extension of the shelter, separating the chambers in the previously indicated cubicles, to measure the amount of radon gas generated in the different areas within the same room. The measurement areas of the study correspond to the following cubicles, as shown in Figure 10.

### 4.2. Plaza Balmis Shelter

**Figure 11:**
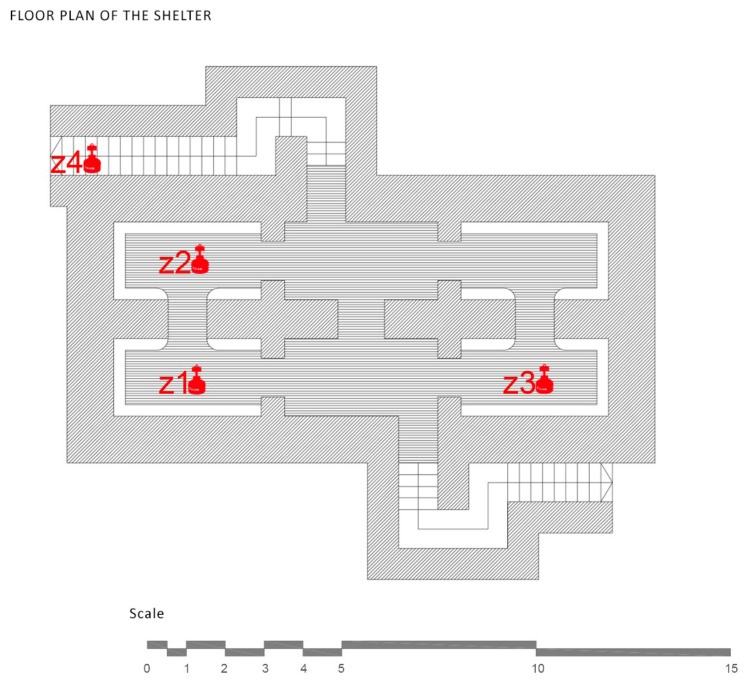
Measurement areas made inside the Plaza Balmis shelter.

The data collection of the shelter in Doctor Balmis Square was conducted for 22 days, starting from March 15, 2016, and it was performed in a homogeneous way over all of the shelter. The measurement zones of the study correspond to the cells shown in Figure 11, with the measurement zones being the following.

The floor plan of the shelter, without complete partitions, allows all of the rooms to be connected and to act in a homogeneous way, with ventilation produced only from the access that is available. The walled access generates a bottleneck where the air is not regenerated as much as in the rest of the refuge. There are also two grilles, on top of the old vents, but they are collapsed and cause humidity problems.

The shelter has similar characteristics to Plaza Seneca’s with respect to measuring the amount of radon generated inside for different reasons: being buried under a square, the low ventilation, the structure, the building materials used and the situation.

### 4.3. Santa Barbara Castle

The air quality study conducted at Santa Barbara Castle lasted four months, due to its uniqueness and extension. The first measurements were taken on 1 December 2015 and the last on 15 March 2016. Two fundamental premises were accounted for when choosing the measurement sites: closed rooms with little ventilation and the possibility of remaining without the transit of people during the days that the measurements were taken.

**Figure 12:**
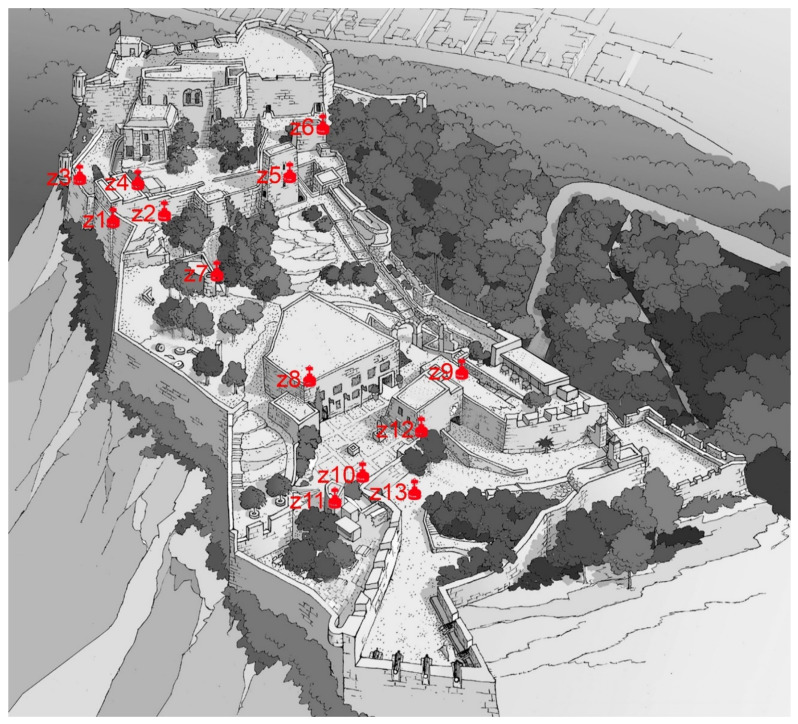
Image of the castle with the locations of the cameras in the studio from an image of the City of Alicante. (Own fountain).

This fortification is an emblematic place in the city of Alicante, which due to its history, has rooms from different periods and therefore different buildings and architectural styles [41]. The castle has been remodelled for different reasons and periods. The rooms measured have different ventilation conditions, which has led to the different results obtained. Figure 12 shows the locations of the rooms studied.

### 4.4. Ereta Powder Keg

The Polvorín de la Ereta is a single room building of 60 m^2^; a single study was conducted for 10 days starting from 23 May 2015, to determine the quality of the indoor air with radon gas as a reference. Four points were chosen for the measurement phase, although they belong to the same room. As seen in the previous sections, the radon concentration can be variable within the same space, which is a fact that has been confirmed by different studies that have dealt with the subject [42,43].

**Figure 13:**
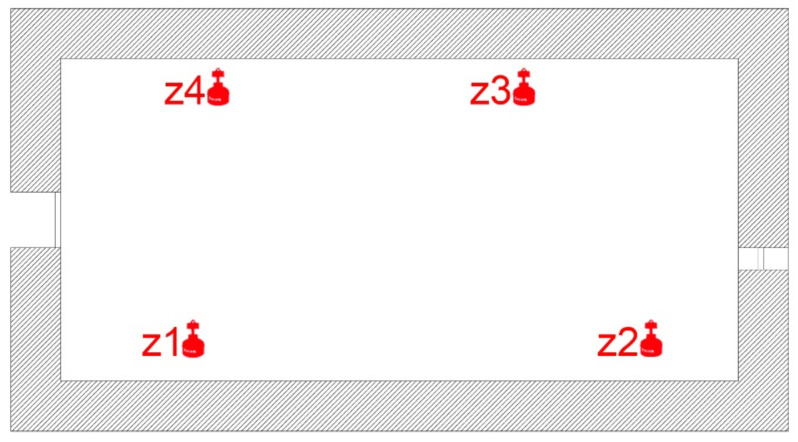
Plant of the Ereta powder keg with the layout of the measurements made in the study.

The measurement areas of the study correspond to the camera arrangement shown in Figure 13.

The construction consists of a single block of reinforced concrete that allows for cross-ventilation between the window and the door (oriented in the NE-SO axis). During the study, the room was completely closed, but the type of carpentry in place makes the air renewal continuous, and thus, it was foreseeable that the results of the amount of radon gas present would be low unless there was some relevant source of emission.

### 4.5. Study of the Air Quality in the Luceros-Marq Tunnel

This study was performed to measure the amount of radon gas inside the Monte Benacantil tunnel, which connects the Marq-Castillo stops with the centre of Alicante and Luceros and will in the future lead to the Renfe stop; it was conducted on 9 and 17 June 2016.

The data collection and process management inside the tunnel were performed with the collaboration of the company FGV, which is in charge of the maintenance of the TRAM in Alicante. For reasons of use, the introduction and collection of the cameras to conduct the study had to be done at midnight because there are safety protocols in which no people can be present when the trains are in motion.

The measurements were used under normal operating conditions inside the tunnel, i.e., the trains were running and the active mechanical ventilation means was present. For this reason, air renewal was high, and the amount of radon gas present was expected to be low, which is an aspect that was intended to be verified.

**Figure 14:**
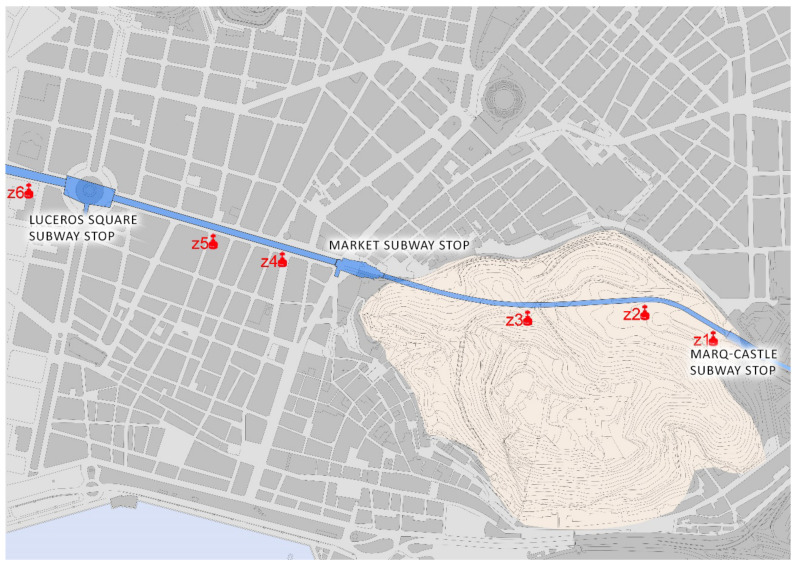
Image of the measuring points inside the tunnel.

The tunnel has an average depth of 15 m and connects by a 1700 m conduit to the existing points of the TRAM stop at the foot of the castle (Marq-Castillo), Mercado and Luceros, with the future Renfe stop that is still being excavated although it is not yet connected to the tracks. The survey points correspond to the following areas inside the tunnel, as shown in Figure 14.

The tunnel, despite being buried 20 m at its deepest point, has a connection to the outside when it exits from the city centre toward the northern area, when it enters Mount Benacantil. This fact, together with the large ventilation systems and the continuous movements of trains, generates a constant renewal of air inside.

### 4.6. Serra Grossa Tunnel

The planning of the study to measure the indoor air quality relative to radon gas as an indicator was conducted in the Serra Grossa tunnel, and it was begun by establishing the construction characteristics of the installation. The three parts that make up the tunnel have been accounted for when planning the areas to be sampled: the tunnel in the mine, the secondary exhaust outlet perpendicular to the main core and the north outlet that is walled up, but it has an upper skylight, which makes it an isolated ventilated area. Therefore, there are three possible air inlets: the north outlet, the exhaust outlet and the south outlet. The tunnel with these structures is blocked off and, therefore, the movement of the air in the interior is very low, with the exception of these three mentioned zones.

**Figure 15:**
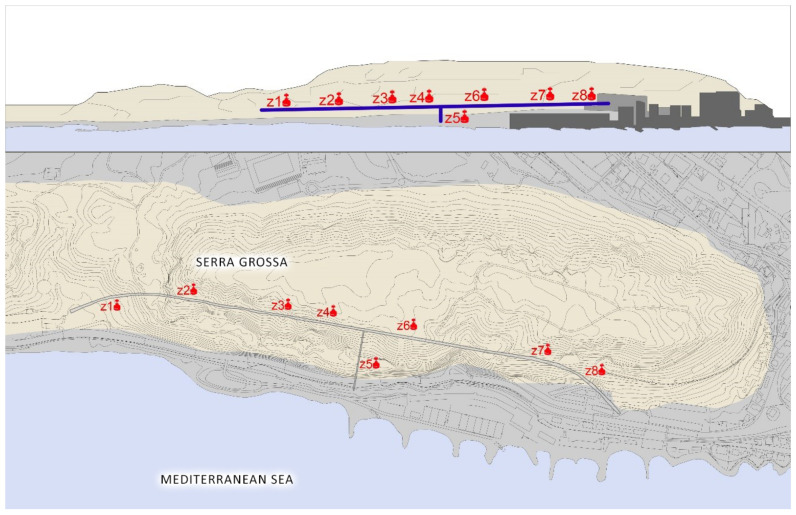
Top image of the tunnel elevation with the arrangement of the measuring elements. The Serra Grossa and the Albufera can be seen. Image below the tunnel floor with the disposition of the measuring elements.

The measurement areas are shown in Figure 15. The design of the tunnel and the circumstances in which it is located have meant that during the 12 days of study, the movement of people and vehicles inside has been minimal, because only the security services guarding the site have been able to pass through.

### 4.7. The Británica’s Deposits

The study conducted inside the old British fuel tanks lasted 2 days, starting from 7 September 2016. The entrance to this place was made through the south door, which is currently partially closed with damaged bars that do not prevent passage. This door was used to access the study. There are other hidden entrances in the mountain that connect with the large galleries, but they are closed and have not been explored during the work in the complex.

**Figure 16:**
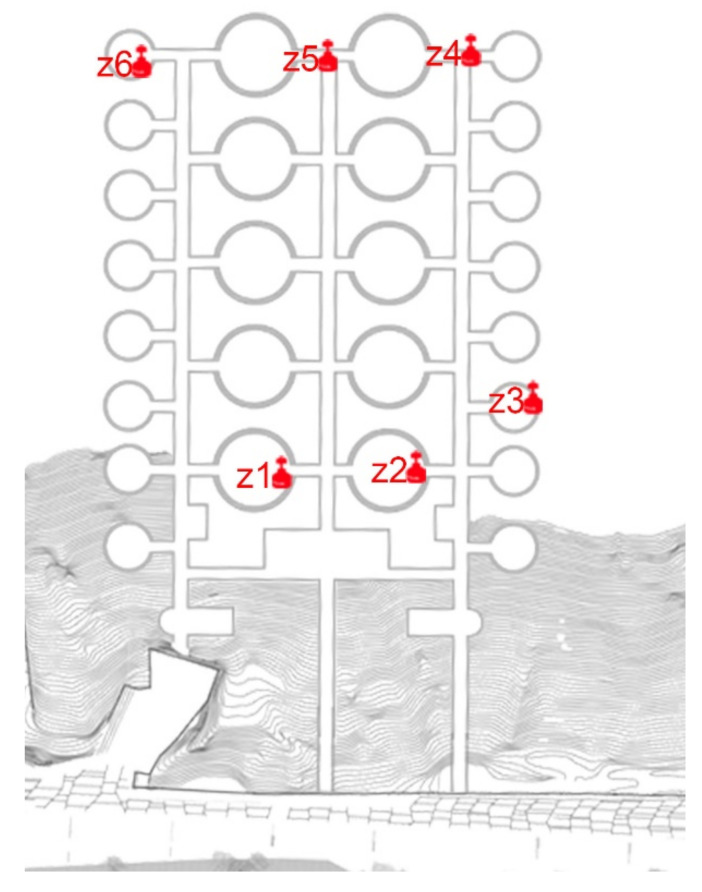
Plan view of the placement of the measurement devices inside the old tanks.

The measurement areas of the study in the former Británica Isles deposits are shown in Figure 16.

The design of this work allows communication among all of the rooms, and the only ventilation comes from the main entrances and from some elements that emerge in the top of the mountain that in the past served the factory to treat the fuel, and thus, the air renewal is almost null.

This old factory is very interesting for the study of the amount of radon gas due to its location in Serra Grossa and the type of construction. In addition, because of its activity, it played a transcendental role in the supply of oil derivatives for the whole country, and its underground structure is the product of engineering work that was conducted more than 60 years ago, having lasted over time despite the fact that the mountain with its domes is almost 20 m high.

## 5. Discussion of Results

The Spanish Mediterranean coast, where the city of Alicante is located, has mainly clayey soils. In the urban area of the city are the two mountains that are compared in this study: Monte Benacantil and Serra Grossa. Therefore, they can be considered to have the same igneous composition.

The ventilation of the interiors of the buildings is fundamental in defining their air quality because the greater the number of renovations (per unit time) is, the lower the number of stale particles in the environment. Therefore, radon gas is used to analyse whether the ventilation is adequate inside the railway tunnels, i.e., whether the area where the extraction machines are located (Monte Benacantil) has better ventilation than the area where the raw material is excavated (Serra Grossa).

Table 1 shows the results obtained in the study, which was conducted in the seven buildings.

In the Plaza Seneca shelter, the maximum amount of radon gas obtained inside the shelter was 151 Bq/m^3^ in zone 2, and therefore, it is only necessary to monitor the amount of radon gas present there from time to time to control that the value does not increase. This finding means that the shelter is sufficiently ventilated, and it is not necessary to conduct remodelling actions at this point to minimise the risks due to this problem. On the other hand, the shelter is currently only part of a museum tour, and therefore, people do not spend much time inside it, and the amounts that could be higher depending on poorer ventilation or those caused at other times of the year would not greatly affect the people who visit the shelter.

The Plaza Balmis shelter has a uniform volume, with all of the cells connected and a single direct air intake from the outside, from the access that is currently in operation. With these conditions, the values obtained range from 96.98 Bq/m^3^ to 224.67 Bq/m^3^ of the walled-in entrance, causing a deviation of 43%. For the standard used, explained above, the recommendations established by different national and international bodies have been integrated. We accounted for the level of 100 Bq/m^3^ as the first threshold to which permanent monitoring of the measurement of the presence of gas corresponds and the level of 300 Bq/m^3^ as the threshold at which corrective ventilation measures must be taken. With these criteria, only monitoring of the amount of radon present should be made for this installation, because all measurements are below the set 300 Bq/m^3^ and almost all are above 100 Bq/m^3^. At present, this area is not in use, and these results must be accounted for in the future, when it is opened to the public; then, the movement inside will be greater, and thus, the air renewal will improve.

Of the thirteen rooms analysed at Santa Barbara Castle, the values that were obtained are well below the limits that should be the criteria for intervention that were found in the following places: the elevator room, the Santa Catalina tower, the Sant Jordi tower, the Bon Repós powder magazine and the cistern. In these areas, air renewal is very high due to the ventilation systems and the movement generated by visitors, whether by mechanical means or by the carpentry shops. The amount of radon gas present in these five places is less than 100 Bq/m^3^, and therefore, improvement is not required in this aspect. However, seven of the rooms analysed obtained average values between the minimum 100 Bq/m^3^ and the maximum 300 Bq/m^3^: the administration area, the corridor of the Raval Roig, the Philip II display case, the oven, the dungeon, the corridor next to the dungeon and the current video room. With the above criterion, only monitoring of the amount of radon present in these rooms should be performed. The results obtained in these rooms are similar to those found in the Plaza Séneca shelter, with values below 300 Bq/m^3^, which is interpreted as the reference value for action and, therefore, does not require taking corrective measures, but it does require monitoring since it is above 100 Bq/m^3^. The room with the highest value was the warehouse next to the administration room, which had values above 300 Bq/m^3^ in all of the measurements made there. Of the 8 measurements made, the average value was 647.09 Bq/m^3^, and these measurements were taken under different conditions of duration. The warehouse is a room of approximately 6 m^2^ without windows and with the only entrance of air coming from the door that connects it with the administration area of the castle, a zone that is eventually ventilated. The exact location of the warehouse is inside the mountain, dug in the rock of the Monte Benacantil, and this room is totally in contact with the land. The exclusive use of storage allows us not to take additional measures, as long as it is not used habitually by people. The results obtained have been communicated to the Town Hall to allow them to know the state of the stay.

The powder magazine building has a single rectangular-shaped volume and two opposite entrances that provide constant airflow from the outside. With these conditions, the values obtained range from 121.32 Bq/m^3^ to 243.45 Bq/m^3^, with a maximum deviation of 45%. We accounted for the level of 100 Bq/m^3^ as the first threshold from which the permanent monitoring of the gas presence measurement corresponds and the level of 300 Bq/m^3^ as the threshold from which corrective ventilation measures must be taken. With these criteria, only monitoring of the amount of radon present should be taken for this installation, because all measurements are below the set 300 Bq/m^3^ and all measurements are above 100 Bq/m^3^. At present, it is in restricted use, and thus, the entry of people is rare.

Of the six areas that were studied inside the Luceros-Marq tunnel, all of the values were much lower than those that could be considered to be capable of action. The only place that stood out, with an average of 182 Bq/m^3^, was the section of the tunnel that links the current Luceros stop with the future Renfe stop (Zone 6). This specific place is under construction, and at the moment, neither the trains nor the air extraction systems are connected; therefore, the amount of renewed air is lower than in the operative sections. This fact will change when the zone becomes operational.

The average radon gas values obtained in the Serra Grossa tunnel range from 694.75 to 1145.46 Bq/m^3^ in the different areas measured. The values obtained in these areas are well above the threshold of 300 Bq/m^3^, which is established in this study as the value above which corrective ventilation measures must be taken. With its completion and implementation, the environmental conditions will change radically, which will allow the corrective measures that can be proposed to coincide with the further plans for the execution of the project.

**Figure 17:**
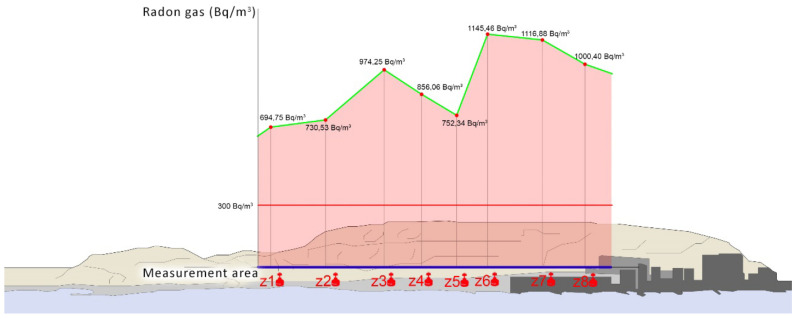
Comparative bar graph of the results obtained in the Serra Grossa tunnel, measured in Bq/m^3^, superimposed on the tunnel elevation.

The results obtained are consistent with the central hypothesis of this research. The areas that have contact with the ground and little ventilation can show, as is the case with this tunnel, quantities of radon gas that are very harmful to health, even in places that were established as low exposure within the Marna map (Figure 17).

The results obtained in the deposits of the Británica show a high average amount of radon gas in its interior, which is explained by the small amount of air movement that is produced and the contact with the ground. The results obtained in the eleven measurement areas varied between 1203.62 and 2413.48 Bq/m^3^, which quadrupled the threshold of 300 Bq/m^3^ in the most favourable case, and 300 Bq/m^3^ was established within this study as the value above which corrective ventilation measures must be taken.

**Figure 18:**
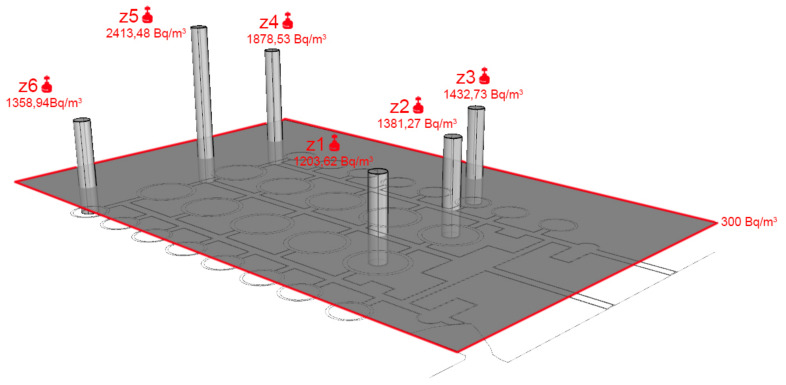
3D graph of the results obtained, measured in Bq/m^3^. This graphic representation shows the complete plan of the building and the values obtained in each of the locations. In the plan, the parts marked in red show a value of 300 Bq/m^3^ or above, and at these locations, measures must be taken to reduce the radon gas.

The old deposits of the Británica have obtained values that are much higher than those estimated for construction in Alicante, if we account for the Marna map, where the Levante area is considered to be low risk [1]. When this area is rehabilitated, the high quantities of radon gas in its interior must be accounted for by adopting solutions. These solutions must be aimed at renewing the interior air more and better by means of mechanical processes, while not making more openings in the mountain that would distort the landscape and weaken the structure. For this reason, without implementing remodelling projects that include adequate ventilation of the facility, the current conditions should not be considered to be suitable for people to stay in this area (Figure 18).

## 6. Conclusions

The Spanish Civil War shelters in the city of Alicante that were analysed present optimum conditions for being open to the public and do not require additional measures for improving the ventilation.

The Luceros-Marq tunnel, which passes through the slopes of Mount Benacantil, has a high level of air renewal in its interior, which is produced by both the movement of trains and by the extraction systems, and the accumulation of radon gas in its interior is low.

In the Santa Barbara Castle, several areas were analysed, of which only the administration warehouse required measures to improve the ventilation, since the accumulation of radon gas in its interior exceeded 300 Bq/m^3^, which is established as a reference level from which to initiate remedial actions in the buildings. Despite being a construction that is in contact with the ground, the Ereta powder magazine does not accumulate large quantities of radon gas inside, and therefore, no improvement measures should be conducted.

The Serra Grossa tunnel, which was in the construction phase and did not have any movement, has reached high values of radon gas in its interior, which have exceeded 300 Bq/m^3^. In the future, when this construction is used for train circulation, it will incorporate mechanical air renewal measures as well as facilitating the movement generated by its activity and will presumably present similar conditions to the Monte Benacantil tunnel.

High values of radon gas were obtained in the interior of the old deposits of the Británica, which is located in the Serra Grossa. These values are quadruple the values for the smaller cases that were over the 300 Bq/m^3^ established reference level. Therefore, in the event of being enabled for the presence of people, measures should be taken to renew the interior air, which should be fundamentally mechanical in an attempt to not damage the landscape.

From the comprehensive research carried out, it follows that in low or very low risk areas within the Marna map of exposure to radon gas, in constructions on limestone rock, with low ventilation and high contact with the ground, high levels of the gas can accumulate. 

## Figures and Tables

**Table 1:** Results obtained for the different measurements that were conducted.

Study	Zone	Place	Number of Samples Chamber-Electret	Type of Measurement	Average Radon Gas Concentration (Bq/m^3^)	Desviation (Bq/m^3^)
	Zone 1	Cubicle 5	8	Short-Short	79.67	7.26
	Zone 2	Cubicle 6	8	Short-Short	151.7	10.2
**Plaza Seneca shelter**	Zone 3	Cubicle 15	8	Short-Short	130.63	11.3
	Zone 4	Cubicle 35	8	Short-Short	63.48	5.7
	Zone 5	Cubicle 22	8	Short-Short	71.04	7.2
	Zone 1	Part 1	6	Short-Short	216.44	22.36
	Zone 2	Part 2	6	Short-Short	116.84	11.2
**Plaza Balmis shelter**	Zone 3	Part 3	6	Short-Short	96.98	9.7
	Zone 4	Secondary entrance	6	Short-Short	224.67	21.8
	Zone 1	Administration warehouse	8	Short-Short	647.09	64.1
	Zone 2	Administration	6	Short-Short	252.63	24.3
	Zone 3	Raval Roig	6	Short-Short	152.66	14.2
	Zone 4	Elevator room	4	Short-Short	65.97	6.25
	Zone 5	Santa Catalina Tower	5	Short-Short	59.25	6.2
	Zone 6	Sant Jordi Tower	6	Short-Short	43.93	4.6
**Santa Barbara Castle**	Zone 7	Gunpowder magazine	5	Short-Short	86.23	8.4
	Zone 8	Felipe II Showcase	6	Short-Short	189	48
	Zone 9	Oven	4	Short-Short	142.25	14.2
	Zone 10	Dungeon	4	Short-Short	235.46	23.4
	Zone 11	Corridor next to the dungeon	4	Short-Short	160.7	15.9
	Zone 12	Video room	4	Short-Short	111.79	11.5
	Zone 13	Cistern	4	Short-Short	35.74	3.3
	Zone 1	Location 1	4	Short-Short	121.32	12.2
	Zone 2	Location 2	4	Short-Short	126.45	13.1
**Ereta Powder keg**	Zone 3	Location 3	4	Short-Short	114.19	12.5
	Zone 4	Location 4	4	Short-Short	243.45	24.8
	Zone 1	65m Marq	6	Short-Short	19.08	1.9
	Zone 2	155m Marq, 610m Market	6	Short-Short	12.63	1.1
	Zone 3	424m Marq, 340m Market	4	Short-Short	25.67	2.6
**Luceros-Marq Tunnel**	Zone 4	105m Market, 420m Luceros	4	Short-Short	19.14	1.9
	Zone 5	265m Market, 270m Luceros	6	Short-Short	20.53	2.3
	Zone 6	66m Luceros, direction Renfe	4	Short-Short	182	18.8
	Zone 1	100m from the south entrance	4	Short-Short	694.75	69.5
	Zone 2	300m from the south entrance	6	Short-Short	730.53	73
	Zone 3	500m from the south entrance	6	Short-Short	974.25	93.2
	Zone 4	600m from the south entrance	6	Short-Short	856.06	82.6
**Serra Grossa Tunnel**	Zone 5	700m emergency exit	4	Short-Short	752.34	74
	Zone 6	800m emergency exit	4	Short-Short	1145.46	106.2
	Zone 7	1300m emergency exit	4	Short-Short	1116.88	102.3
	Zone 8	1400m emergency exit	4	Short-Short	1000.4	99.2
	Zone 1	Dome 1	6	Short-Short	1203.62	110.6
	Zone 2	Dome 2	6	Short-Short	1381.27	120.3
**Británica deposits**	Zone 3	Dome 3	6	Short-Short	1432.73	135.7
	Zone 4	End of Aisle 1	6	Short-Short	1878.53	156.2
	Zone 5	Aisle 2	6	Short-Short	2413.48	237.2
	Zone 6	Dome 4	6	Short-Short	1358.94	152.8
